# A novel TSC1 frameshift mutation c.1550_1551del causes tuberous sclerosis complex by aberrant splicing and nonsense‐mediated mRNA degradation (NMD) simultaneously in a Chinese family

**DOI:** 10.1002/mgg3.1410

**Published:** 2020-07-31

**Authors:** Cong Qiu, Chengyan Li, Xiaoyun Tong, Luoyang Dai, Wenda Liu, Yulie Xie, Qimei Zhang, Guohua Yang, Tao Li

**Affiliations:** ^1^ Department of Neurology Renmin Hospital of Wuhan University Wuhan Hubei China; ^2^ Demonstration Center for Experimental Basic Medicine Education of Wuhan University Wuhan Hubei China; ^3^ Central hospital of Yichang City The First Clinical Medical College of Three Gorges University Yichang Hubei China

**Keywords:** frameshift mutation, MINI‐gene, NMD, PTC, *TSC1*, tuberous sclerosis complex, UPF1

## Abstract

**Background:**

Tuberous sclerosis complex (TSC), belongs to autosomal dominant genetic disorder, which affects multiple organ systems in the body, including the skin, brain, lungs, kidneys, liver, and eyes. Mutations in *TSC1* or *TSC2* was proved to be associated with these conditions.

**Methods:**

Gene‐panel Sequence of NGS was used to detect the mutation in a Chinese family. The research further investigates whether aberrant splicing and nonsense‐mediated mRNA degradation (NMD) could serve as a mechanism cause by *TSC1* mutation. MINI‐Gene assay apply by pcMINI‐*TSC1*wt/mut plasmids delivered in HeLa and 293T cell lines. Recombinant plasmids expressing wild‐type and mutant‐type EGFP‐*TSC1* were constructed and transiently transfected into human embryonic kidney cells 293T by lipofectamine. Real‐time PCR and Western Blot were performed to analyze the expression of mRNAs and proteins of EGFP‐*TSC1* and NMD factor UPF1.

**Results:**

The gene test verified a novel heterozygous *TSC1* frameshift mutation (*TSC1* c.1550_1551del) in the proband and her mother. From MINI‐Gene assay, the agarose gel showed that both the mutant and wild‐type mRNA possess two main bands, indicating two splicing modes, named band A and B, respectively. The mutation c.1550_1551del has not produced new splicing site, but there is a selective splicing in varying degree significantly after mutation. On the contrary, function validation assay showed that cells transfected with the mutant *TSC1* plasmids expressed significantly lower *TSC1* in mRNAs and proteins levels, compared with the wild‐type *TSC1* plasmid transfection. A translation inhibitor cycloheximide and small interfering RNA of UPF1 (siRNA‐UPF1) increased mRNA or protein expression of *TSC1* significantly in cells transfected with the mutant plasmids.

**Conclusion:**

Our study demonstrated that the novel *TSC1* frameshift mutation (*TSC1* c.1550_1551del) trigger aberrant splicing and NMD simultaneously, causing decrease of hamartin, then, leading to tuberous sclerosis complex formation.

## Graphical abstract

This paper verified a novel heterozygous TSC1 frameshift mutation（TSC1 c.1550_1551del）in a Chinese family of tuberous sclerosis complex. MINI‐Gene and function validation assay demonstrated that the novel TSC1 mutation triggered aberrant splicing and NMD simultaneously, caused decrease of hamartin, then resulted in tuberous sclerosis complex.

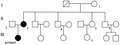

## 1 INTRODUCTION

Tuberous sclerosis complex (TSC) is an extremely multisystemic disease evolved in multiple organs and characteristic by benign tumors in the skin, brain, kidneys, lung, and heart. Loss of function in either *TSC1* (OMIM accession number #605284) or *TSC2* (OMIM accession number #613254) was proved associated with this syndrome. These two genes was identified to be located at chromosomes 9q34 (*TSC1*) and 16p13 (*TSC2*), encoding hamartin and tuberin separately (The European Chromosome 16 Tuberous Sclerosis Consortium, 1993; Van Slegtenhorst et al., 1997). Tuberin and hamartin were found expressed in various human epithelia, such as bronchial, gut, glandular, and renal tubular epithelia (Johnson, Kerfoot, Bushnell, Li, & Vinters, 2001). These two proteins function together with TBC1D7 to form a tuberin‐hamartin‐TBC1D7 complex by coiled‐coil to restrain mammalian target of rapamycin (mTOR)‐mediated signaling pathway and insulin signaling pathway, thus, playing a role in the regulation of both cell growth, proliferation and differentiation (Dibble et al., 2012; Rosner, Hofer, Kubista, & Hengstschläger, 2003; Tee et al., 2002; Van Slegtenhorst et al., 1998). Recent studies have reported that mutations occur more frequently in *TSC2* than in *TSC1*, either in familial or sporadic cases, including a broad spectrum of small insertions and deletions, single‐base substitutions resulting in nonsense codons, missense changes, or variant spliceosomes, and larger genomic deletions (Dabora et al., 2001; Mi, Wang, Jiang, Sun, & Wang, 2014). In some cases, no transcript mRNA was detected with a nonsense mutation, implying that nonsense‐mediated mRNA degradation may be involved in the pathogenesis of TSC (Crino, Aronica, Baltuch, & Nathanson, 2010).

In this study, we detected a novel *TSC1* frameshift mutation c.1550_1551del (p.Arg517GlnfsTer17) from two members of a Chinese family diagnosed as tuberous sclerosis. Our research indicated that the *TSC1* mutation c.1550_1551del triggered aberrant splicing and NMD simultaneously, resulting in decrease in hamartin for the development of the tuberous sclerosis complex.

## 2 MATERIALS AND METHODS

### 2.1 Study subjects

**Figure 1:**
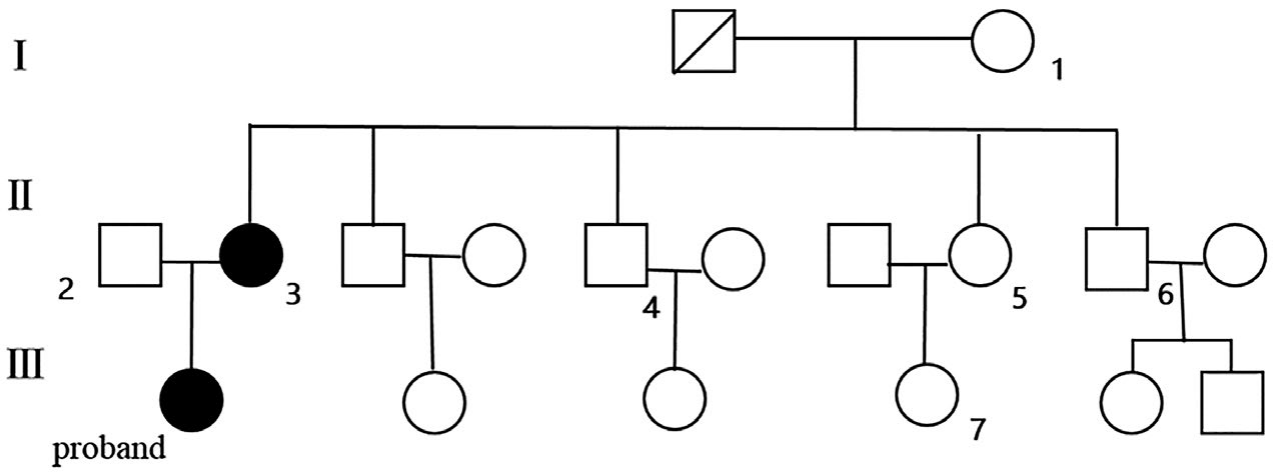
The pedigree and sample collection of the family

The proband and other six members from the family was recruited this study (Figure 1). Magnetic resonance imaging (MRI), ultrasound (US), and computed tomography (CT) were performed to detect the tumorous lesions. The diagnosis made mainly according with updated diagnostic criteria from 2012 International Tuberous Sclerosis Complex Consensus Group (Northrup et al., 2013). Peripheral blood samples from these eight candidates were collected, and *TSC1* gene (GenBank ID: NG_012386.1) for each one was amplified and sequenced. This research was approved by the institutional ethics committee of the Renmin Hospital of Wuhan University.

### 2.2 Analysis of TSC gene

Blood DNA Mini kit (purchased from Simgen Biotechnology, Hangzhou, China) was used for extracting genomic genes from peripheral blood samples. The Gene‐panel Sequence of NGS was used to detect the mutations. So the special target gene acquisition library was constructed by PCR and the products were purified and quantified. Next using the illumina NextSeq500 sequencing machine sequencing, got off the raw data, and converted it into recognizable base sequence by CASAVA (1.8.2) software, then, commented mutations by biological information analysis, and picked out the mutant site according with the clinical manifestation, then, proceeded Sanger sequencing for pedigree validation. This part of the work was completed commercially by Peking Kangxu Medical Laboratory Company of China.

### 2.3 Construction of pcMINI‐gene and transcription analysis

Using the genomic DNA as template, the wild‐type *TSC1* gene fragment was obtained by nested PCR. A pair of primers was designed to introduce the deletion mutation. The mutant‐type *TSC1* was amplified by overlap extension PCR. The primer sequences are listed as Table 1.

**Table 1:** The primers used in MINI‐Gene essay

Name	Primer sequence (5′−3′)
*TSC1*‐37525‐F	gtattctgacttgactatatc
*TSC1*‐39957‐R	ctgtgttgttagcttaacacag
*TSC1*‐37892‐F	gtaatgtatgtgggattgctatg
*TSC1*‐39618‐R	tccccaagcacctgtaaagtag
pcMINI‐*TSC1*‐Kpn1‐F	ggtaGGTACCcacctcagcctcctgagtagctg
pcMINI‐*TSC1*‐BamH1‐R	TAGTGGATCCcttgcagagggcacatatgaaag

**Figure 2:**
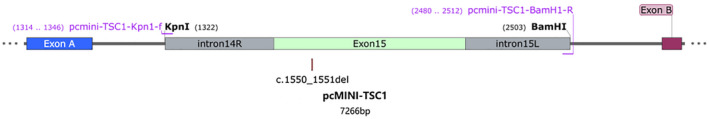
The minigene plasmid profile of pcMINI‐*TSC1*

The restriction enzymes Kpn I and EcoR I were used to digest the PCR products, then, the products were ligated into the same restriction sites of pcDNA 3.0+ (purchased from Bioeagle Biotech Company, Ltd., Wuhan, China) to produce the recombined plasmids pcMINI‐*TSC1*‐wt/mut (Figure 2).

After overnight ligation at 4℃, the recombinant plasmid was transformed into *E*.* coli* competent cells *E*.*coli* DH5α. After 12 hr of incubation, colony PCR was performed, using pcMINI‐*TSC1*‐KpnI‐F and pcMINI‐*TSC1*‐BamHI‐R as primers, to verify the construction of the required base changes. Plasmid sequencing was also conducted to confirm the introduction of the mutation. TIANprep Mini Plasmid Kit (purchased from TIANGEN BIOTECH, Beijing, China) was used for plasmid extraction according to the instruction.

HeLa cells were cultured in DMEM + 10% FBS. The recombinant plasmids were transiently transfected into HeLa cells by Liposomal Transfection Reagent (purchased from Yeasen Biotech Company Limited, Shanghai, China) according to the instruction.

Forty‐eight hours after transfection, RNA was extracted by the “single‐step” method (Chomczynski & Sacchi, 2006) and reverse‐transcribed into cDNA using the genome DNA PrimeScript™ RT reagent Kit (purchased from Takara Biomedical Technology, Beijing, China). The cDNA was amplified by PCR and analyzed by agarose gel electrophoresis. The primers used were *TSC1*‐37525‐F of 5′ end and *TSC1*‐39957‐R of 3′ end.

### 2.4 Plasmid construction and transfection

A DNA fragment containing *TSC1* cDNA full length was obtained by gene synthesis. The p3XFLAG‐CMV‐7.1‐*TSC1*‐wt fragment (3,519 bp) was amplified by PCR using the DNA fragment obtained by gene synthesis as a template, while the pEGFP‐C1‐*TSC1*‐wt fragment (3,515 bp) was amplified in the same way. Plasmid pEGFP‐C1 and plasmid p3XFLAG‐CMV‐7.1 was purchased from the Bioeagle Biotech Company, Ltd., Wuhan, China. The p3XFLAG‐CMV‐7.1‐*TSC1*‐mut and pEGFP‐C1‐*TSC1*‐mut containing c.1550_1551del was amplified by overlap extension PCR. Restriction sites introduced by primers. The primers sequences are listed as Table 2.

**Table 2:** The primers used in vector construction

Name	Primer sequence (5′−3′)
GFP‐*TSC1*‐Kpn1‐F	CGACGGTACCATGGCCCAACAAGCAAATGTCGGG
GFP‐*TSC1*‐Kpn1‐R	gactggtaccTTAGCTGTGTTCATGATGAGTCT
CMV‐*TSC1*‐Not1‐F	gcttgcggccgcCATGGCCCAACAAGCAAATGTCG
CMV‐*TSC1*‐Kpn1‐R	gactggtaccTTAGCTGTGTTCATGATGAGTCT
*TSC1*‐mut‐F	TCCCAGGTTCTCAGCAAGACCCACTCGGCAGC
*TSC1*‐mut‐R	GCTGCCGAGTGGGTCTTGCTGAGAACCTGGGA

**Figure 3:**
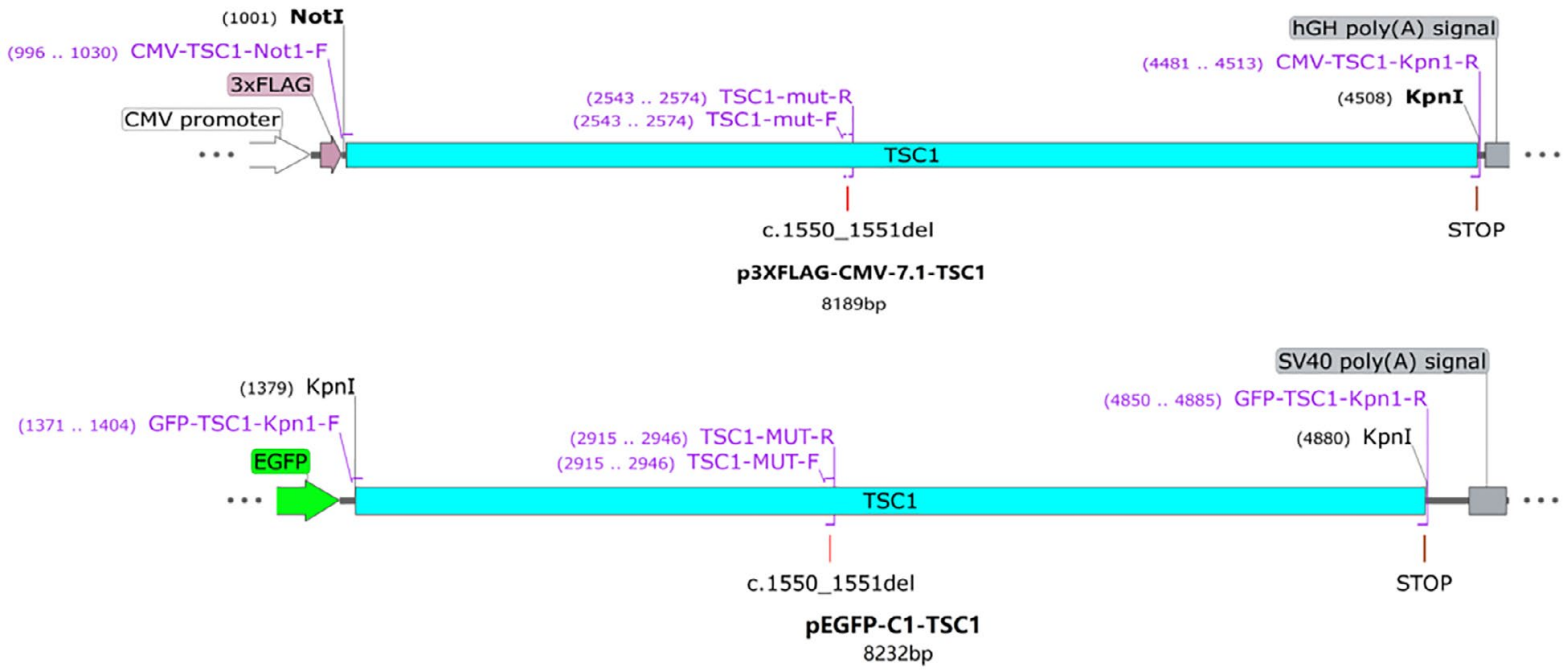
The strategy of vector construction: Using the DNA fragment of gene synthesis as a template, the primers CMV‐TSC1‐Not1‐F and CMV‐TSC1‐Kpn1‐R were designed to amplify the p3XFLAG‐CMV‐7.1‐TSC1‐wt fragment (3,519 bp), GFP‐TSC1‐Kpn1‐F and GFP‐TSC1‐Kpn1‐R amplified the pEGFP‐C1‐TSC1‐wt fragment(3,515 bp). A pair of mutant primers TSC1‐mut‐F and TSC1‐mut‐R were designed. Fragment 1(1,579 bp) was amplified by using CMV‐TSC1‐Not1‐F and TSC1‐mut‐R as primers, and fragment 2 (1,971 bp) was amplified by using TSC1‐mut‐F and CMV‐TSC1‐Kpn1‐R as primers. Using a 1:1 mixture of Fragment 1 and Fragment 2 as a template, fragment p3XFLAG‐CMV‐7.1‐TSC1‐mut containing the mutation c.1550_1551del was obtained by overlap extension PCR using CMV‐TSC1‐Not1‐F and CMV‐TSC1‐Kpn1‐R as primers. And the fragment pEGFP‐C1‐TSC1‐mut was amplified in the same way by overlap extension PCR. (Purple font and logo indicate primer name, orientation, and location)

The PCR condition was 30 cycles, 98°C for 10 s, 57°C for 30 s, 72°C for 30 s. The PCR products were digested with Kpn I and EcoR I of the restriction enzymes, then, ligated into the same restriction sites of pEGFP‐C1/p3XFLAG‐CMV‐7.1 to produce the recombinant plasmids, pEGFP‐C1‐*TSC1*‐wt/mut and p3XFLAG‐CMV‐7.1‐*TSC1*‐wt/mut (Figure 3).

The recombinant plasmid was ligated overnight at 4°C and transformed into *E*.* coli* competent cells *E*.* coli* DH5α. After 12 hr of incubation, colony PCR was performed to verify the construction of the required base changes. Plasmid sequencing was conducted to confirm the introduction of the mutation. TIANprep Mini Plasmid Kit (purchased from TIANGEN BIOTECH Company, Beijing, China) was used for plasmid extraction according to the instruction.

Human embryonic kidney 293T cells were cultured in DMEM + 10% FBS + 1% penicillin/streptomycin at 37°C and 5% of CO_2_. Cells were transfected with a pEGFP‐C1‐*TSC1*‐wt/mut or p3XFLAG‐CMV‐7.1‐wt/mut using Liposomal Transfection Reagent (purchased from Yeasen Biotech Company Limited, Shanghai, China) according to the instruction. After 6 hr of transfection, the medium was replaced with fresh complete DMEM culture medium, and the cells were cultured further for 48 hr. Total RNA and proteins were extracted and checked by qPCR and Western blot.

### 2.5 Real‐time qPCR

Total RNA exacted by the “single‐step” method was reverse‐transcribed into cDNA using the PrimeScript™ RT reagent Kit (purchased from Takara Biomedical Technology, Beijing, China) according to the instruction. Real‐time PCR was performed using SYBR Green Mix (purchased from TOYOBO BIOTECH Company Limited, Shanghai, China). Two pairs of primers were designed before and after the mutation site to detect the expression of *TSC1*‐wt and *TSC1*‐mut. The primers before the mutation site was named *TSC1*‐qPCR‐1, and the primers after the mutation site was named *TSC1*‐qPCR‐2. The primer sequences are listed as Table 3.

**Table 3:** The primers used in qPCR

Name	Primer sequence (5′−3′)
UPF1‐s	AGAGGTGACCCTGCACAAGG
UPF1‐as	AGCCGAGGAGGAAGACGTTG
*TSC1*‐qPCR‐1F	TGTTGTGATCGAGTGTGCCA
*TSC1*‐qPCR‐1R	AACAACATCAGCCGAGACGT
*TSC1*‐qPCR‐2F	CCTCCGAGACCAGTTGCTTT
*TSC1*‐qPCR‐2R	ACCATAGTGTCACGCTGCTC

### 2.6 Translation inhibition

In order to inhibit protein translation, the constructed wild‐type and mutant‐type eukaryotic recombinant expression vectors were transiently transfected into 293T cells for 40 hr, and then, treated with cycloheximide CHX (20 ug/ml) for 8 hr. RNA and proteins were extracted and analyzed by qPCR and Western Blot.

### 2.7 RNA interference

HEK 293T cells were further transfected with 50 nmol/ml of UPF1 siRNA and 2.5 μg of a mutant or a wild‐type pEGFP‐C1‐*TSC1* using lipofectamine 2000. After 48 hr of incubation, total RNA and proteins were extracted, and analyzed by qPCR and Western Blot.

### 2.8 Fluorescence microscopy

Transfected HEK 293T cells were cultured on a 6‐well plate, and the expression of pEGFP‐C1‐*TSC1* was examined by fluorescence microscopy under 475 nm blue light irradiation.

### 2.9 Western Blot

The total proteins were extracted from HEK 293T cells after 48 hr of culture. Protein concentration was determined using BCA Protein Quantification Kit (purchased from Yeasen Biotech Company Limited, Shanghai, China) under the guidance of the instruction. 10 μg of proteins were separated on 10% of SDS–PAGE and transferred electrophoretically to the nitrocellulose membrane. Proteins were probed by monoclonal anti‐GFP, anti‐UPF1, or anti‐β‐actin antibody overnight.

## 3 RESULTS

### 3.1 Clinical manifestations and imaging findings

**Figure 4:**
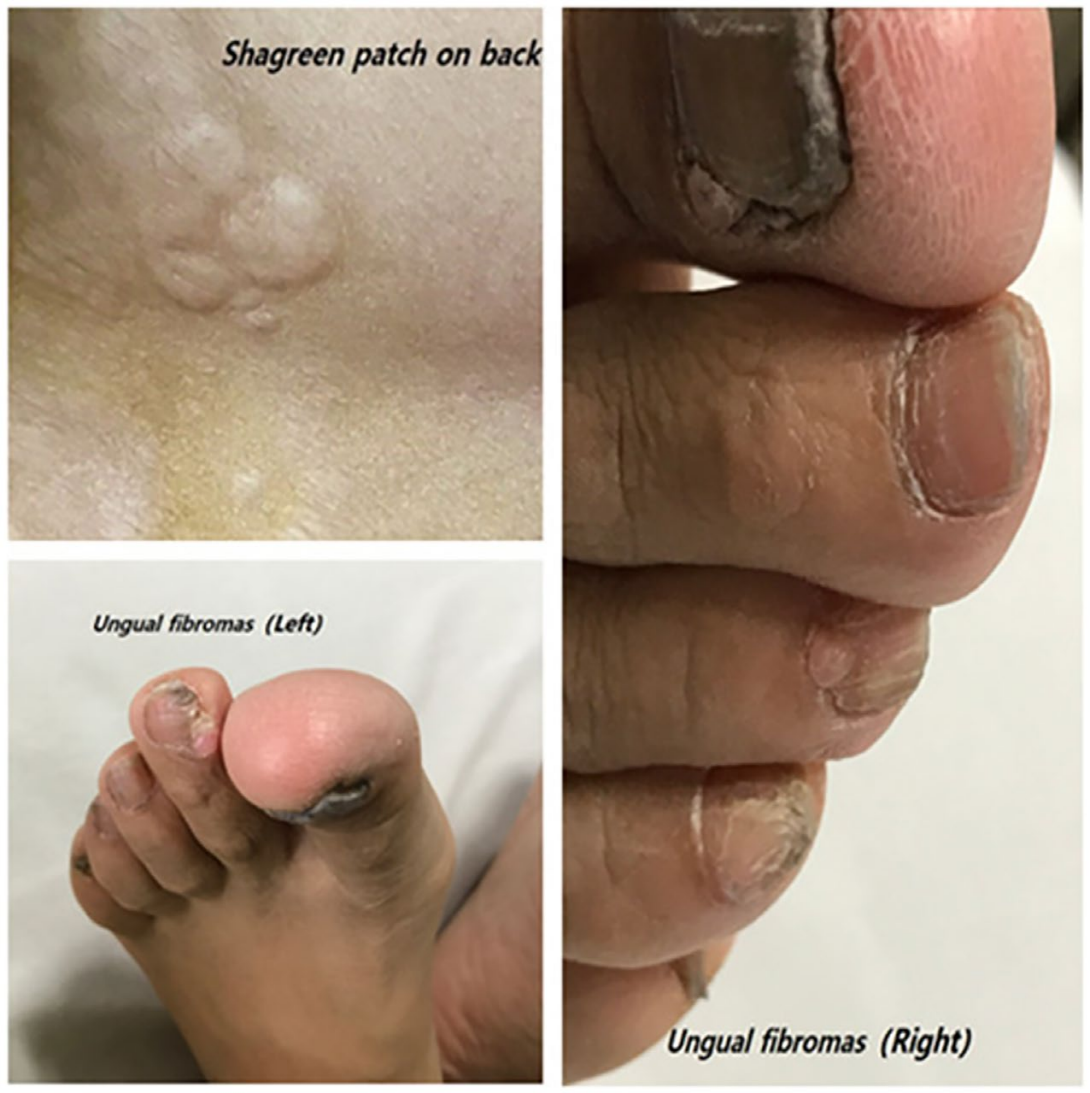
Skin lesions and ungual fibromas in the proband

**Figure 5:**
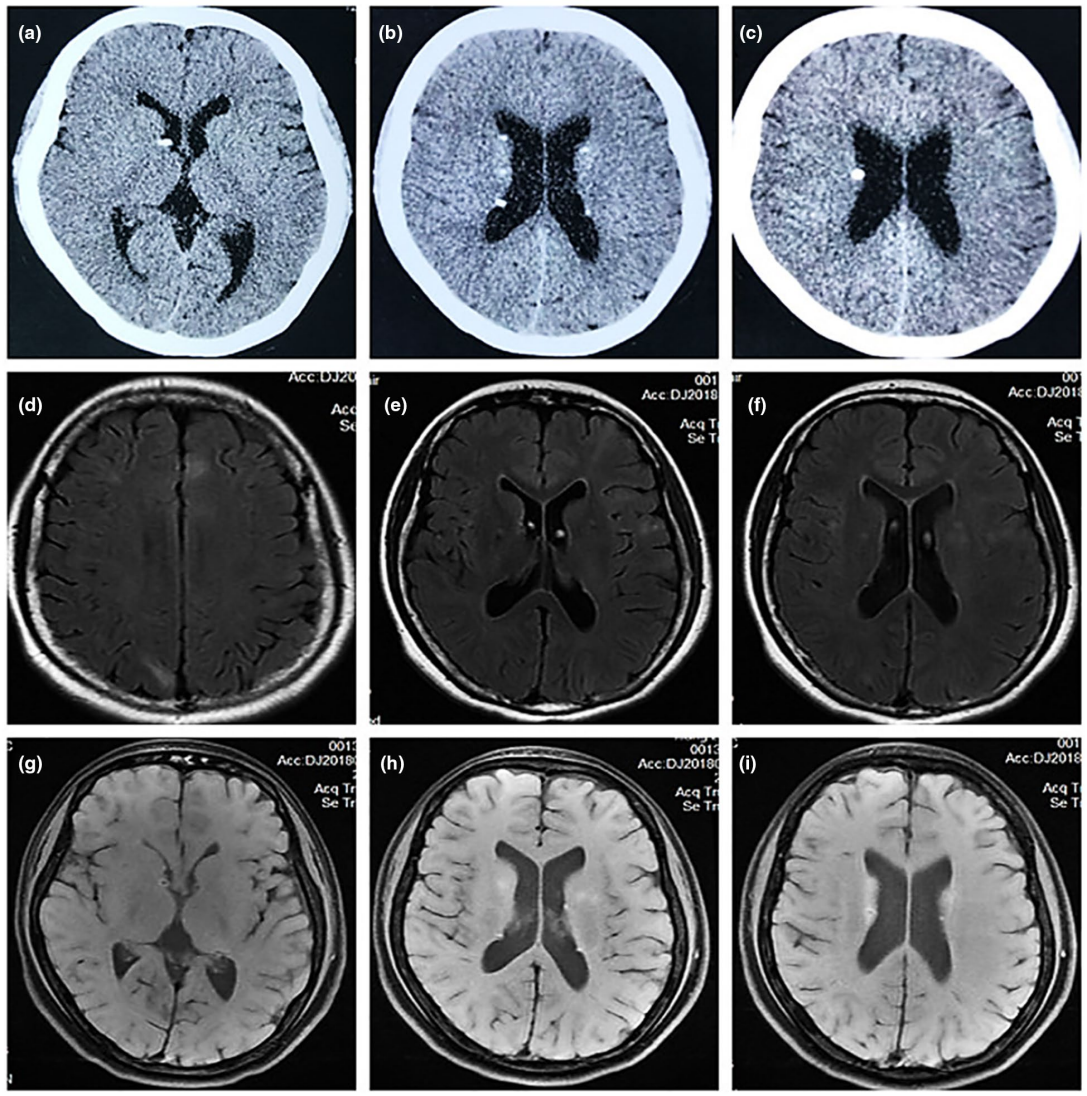
Imaging appearance of the Central Nervous System. (a–c) Axial computed tomography of the brain, demonstrating subependymal nodules. (d–f) Axial magnetic resonance imaging (MRI) (T2 flair) of the brain, demonstrating the cortical dysplasia (tubers and radial migration lines). (g–i) Axial MRI (T1 + contrast) of the brain, demonstrating the subependymal nodules

The proband suffering from this disease was a 23‐year‐old female, whose main manifestations were memory deficits and psychotic disorders from the age of 21. Physical examination revealed Shagreen patch on her back and ungual fibromas on her feet (Figure 4). Subependymal nodules have been detected by CT and MRI (Figure 5), but no tumorous lesions found by ultrasound in heart, liver, and kidney. Definite diagnosis made according to the Clinical diagnostic criteria. Her mother, who presented with milder clinical symptoms, was found to have angiomyolipoma in right renal by ultrasound and bilateral subependymal nodules by CT.

### 3.2 DNA sequence

**Figure 6:**
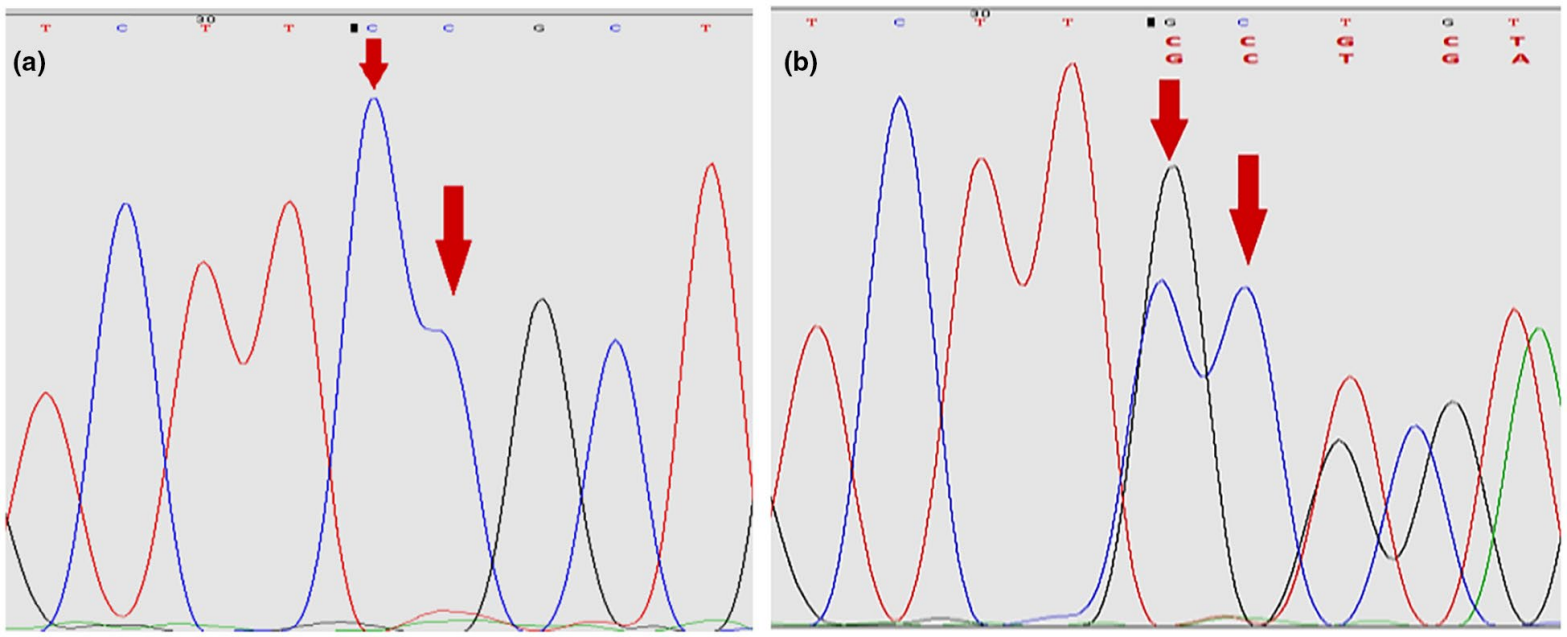
Identification of mutations (c.1550_1551del) in *TSC1* gene. Sequence chromatograms of de novo TSC1 mutation. Diagram (a) and (b) display normal sequences and mutated sequences, respectively. The c.1550_ 1551del (p.Arg517GlnfsTer17) mutation site is indicated by the arrows)

A heterozygous *TSC1* frameshift mutation c.1550_1551del (p.Arg517GlnfsTer17) on Exon 15 was detected from the proband and her mother, which has never been reported on Human Gene Mutation Database (HGMD) (Figure 6). This mutation results in the premature termination codon (PTC) at the position of 17 codons after the mutation site. Genetic analysis among the proband's father and other family members indicated that no mutation in any TSC‐associated genes.

### 3.3 Increase of aberrant splicing in pcMINI‐TSC1‐mut

**Figure 7:**
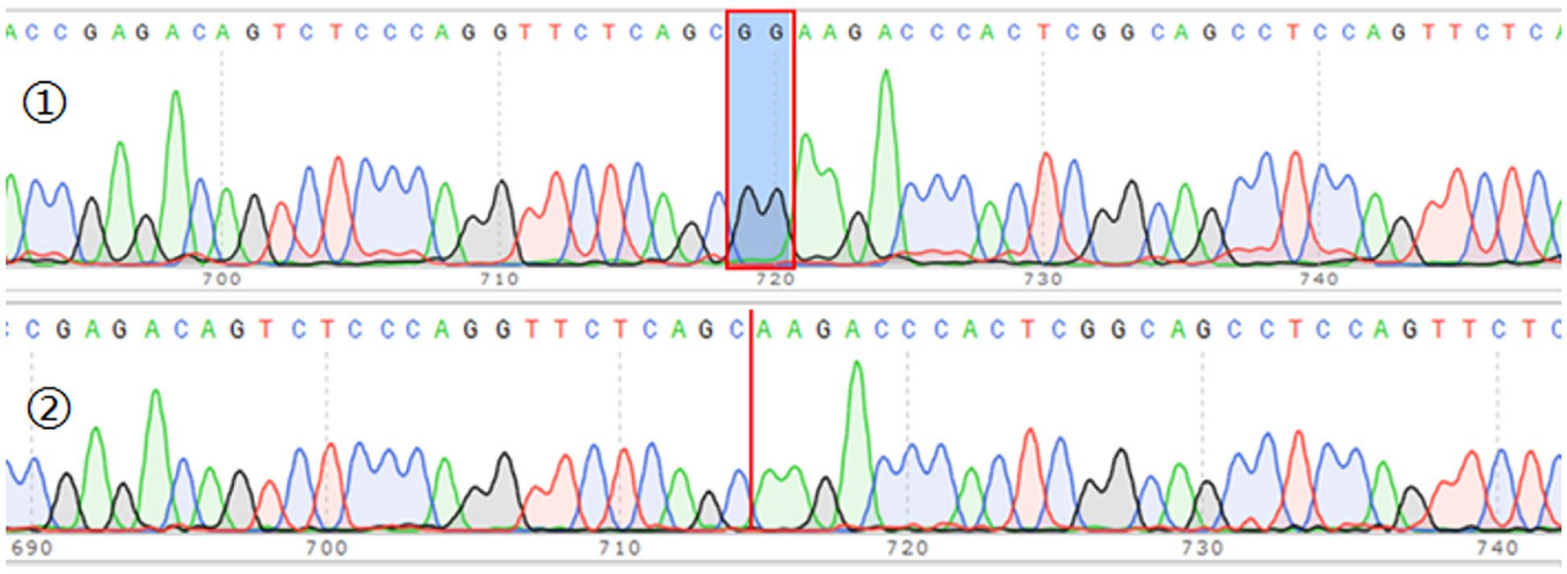
Introduction of mutations into recombinant plasmids. (The colony PCR product was analyzed by agarose gel electrophoresis, and obtained several bands as a result. All bands were sequenced and the results revealed that both wild‐type and mutant‐type minigenes were successfully introduced into the corresponding vector. The sequencing results of wild‐type and mutant‐type minigene are shown as diagrams ① and ②.)

**Figure 8:**
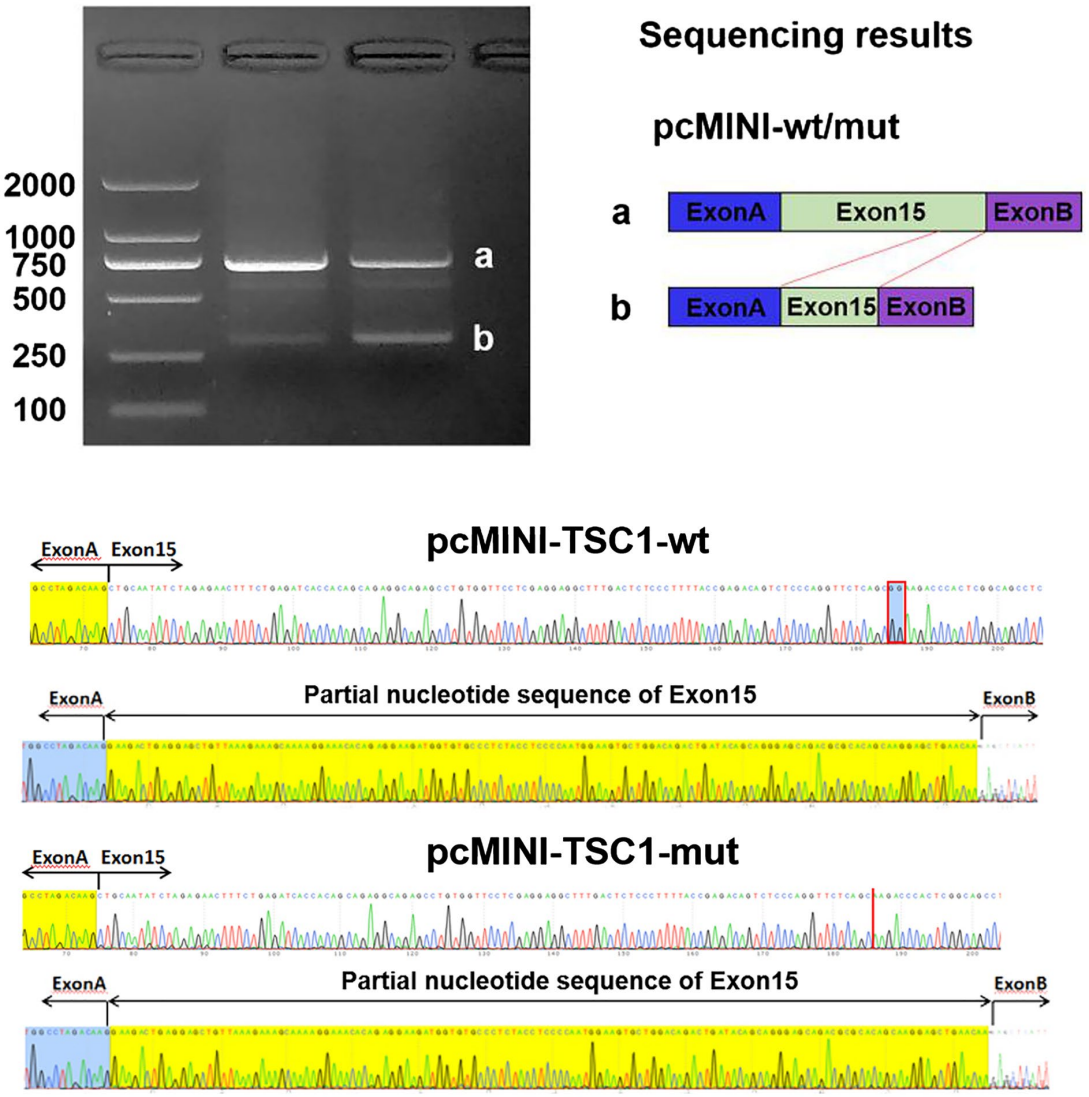
No new splicing site but increase of aberrant splicing in pcMINI‐*TSC1*‐mut. Illustration of two splicing modes of pcMINI‐TCS1‐wt/mut. The diagrams above and below represent the sequence of band A and band B in pcMINI‐TSC1‐wt/mut, respectively. The band A which contains the entire sequence of Exon 15 was considered as expected normal splicing. While band B was considered as a partial deletion of Exon 15 (with 427 bp deleted)

In order to investigate whether the mutation produces a new splicing mode, the mutant‐type and wild‐type pcMINI‐*TSC1* vectors were transfected into HeLa cells. The colony PCR and Recombinant plasmid sequencing revealed that both wild‐type and mutant‐type minigenes were successfully introduced into the corresponding vector (Figure 7). The total RNA was extracted and reverse‐transcribed into cDNA after transfection over 48 hr. The cDNA was amplified by PCR and analyzed by agarose gel electrophoresis. Agarose gel electrophoresis showed that both the wild‐type and the mutant‐type mainly contained two bands, named A and B, respectively. These two bands of the wild and mutant‐type were of the same size, but band A was extremely bright and the band B was weak in the wild‐type, indicating that band A is the main variant. The mutant band A and band B are similar in brightness, and the mutant band B is brighter than the wild‐type band B. Band A and B of both the wild‐type and mutant‐type were sequenced. The result showed that there is no difference between the wild‐type and mutant‐type in splicing modes of neither band A nor band B. It indicates that there are two kinds of splicing modes even in the wild‐type, namely, retaining the complete Exon 15 and retaining part of Exon 15, but the first splicing mode is the main form. The mutant c.1550_1551del has not introduced new splicing site, but there is a selective splicing in varying degree significantly after mutation, the proportion of B‐splicing mode is obviously increased, which reveals that this mutation may be a crucial mechanism for the pathogenesis (Figure 8).

### 3.4 Expression of *TSC1* mRNA and protein in transfected cells

**Figure 9:**
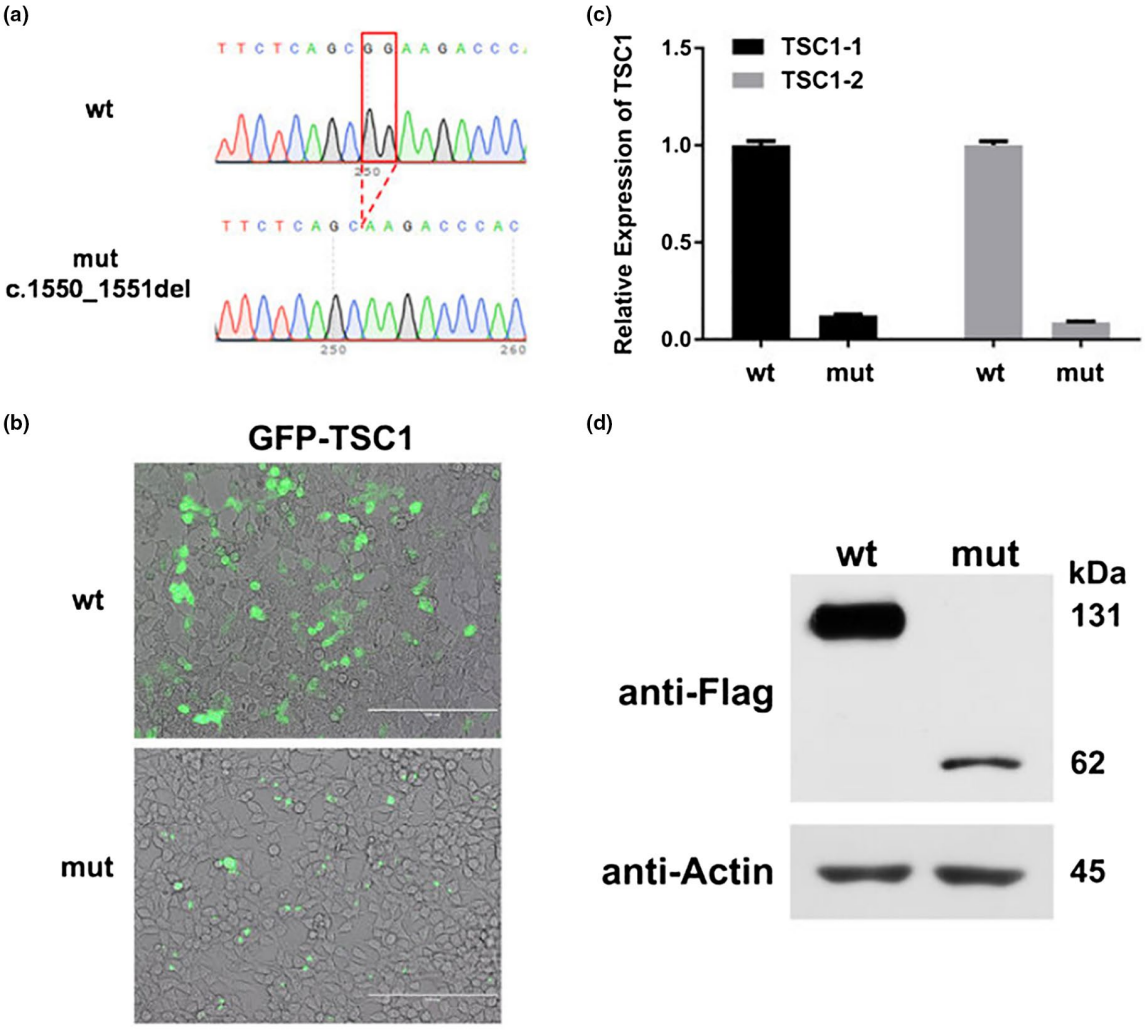
Expression of *TSC1* mRNA and protein in transfected cells. (a) Sequencing results showed that the mutation c.1550_1551del was successfully introduced. (b) Fluorescence microscopy showed that the GFP fluorescent signal in the cells transfected with the mut plasmid was significantly weaker than that of cells transfected with the wt plasmid. (c) The mRNA transcribed from pEGFP‐C1‐TSC1‐mut was significantly lower than that from the wt plasmid, and the mRNA levels expressed by the mutant plasmid were 12% (detected by the primer TSC1‐1) or 8% (detected by the primer TSC1‐2) of the wt plasmid. (d) Expression of mut and wt p3XFLAG‐CMV‐7.1‐TSC1 proteins was detected by Western blot, and the result showed that the protein translated by the cells transfected with the mut plasmid (~62 kDa) was significantly lower compared with which translated by the cells transfected with the wt plasmid (~131 kDa)

To investigate the mutation c.1550_1551del on *TSC1* expression, the plasmid expressing mutant pEGFP‐C1‐*TSC1* cDNA were transfected into HEK 293T cells. After 48 hr of transfection, the mRNA transcribed from pEGFP‐C1‐*TSC1*‐mut was lower than that from the wild‐type plasmid significantly, and the mRNA levels expressed by the mutant plasmid were 12% (detected by the primer *TSC1*‐1) or 8% (detected by the primer *TSC1*‐2) of the wild‐type plasmid (Figure 9A,C). Fluorescence microscopy showed that the GFP fluorescent signal in the cells transfected with the mutant plasmid was significantly less than that of the cells transfected with the wild‐type plasmid. The Western Blot revealed that the protein translated by the cells transfected with the p3XFLAG‐CMV‐7.1‐*TSC1*‐mut was a truncated protein (~62 kDa), whose expression was also significantly lower compared with the wild‐type group (Figure 9B,D).

### 3.5 Degradation of the mutant mRNAs by NMD

**Figure 10:**
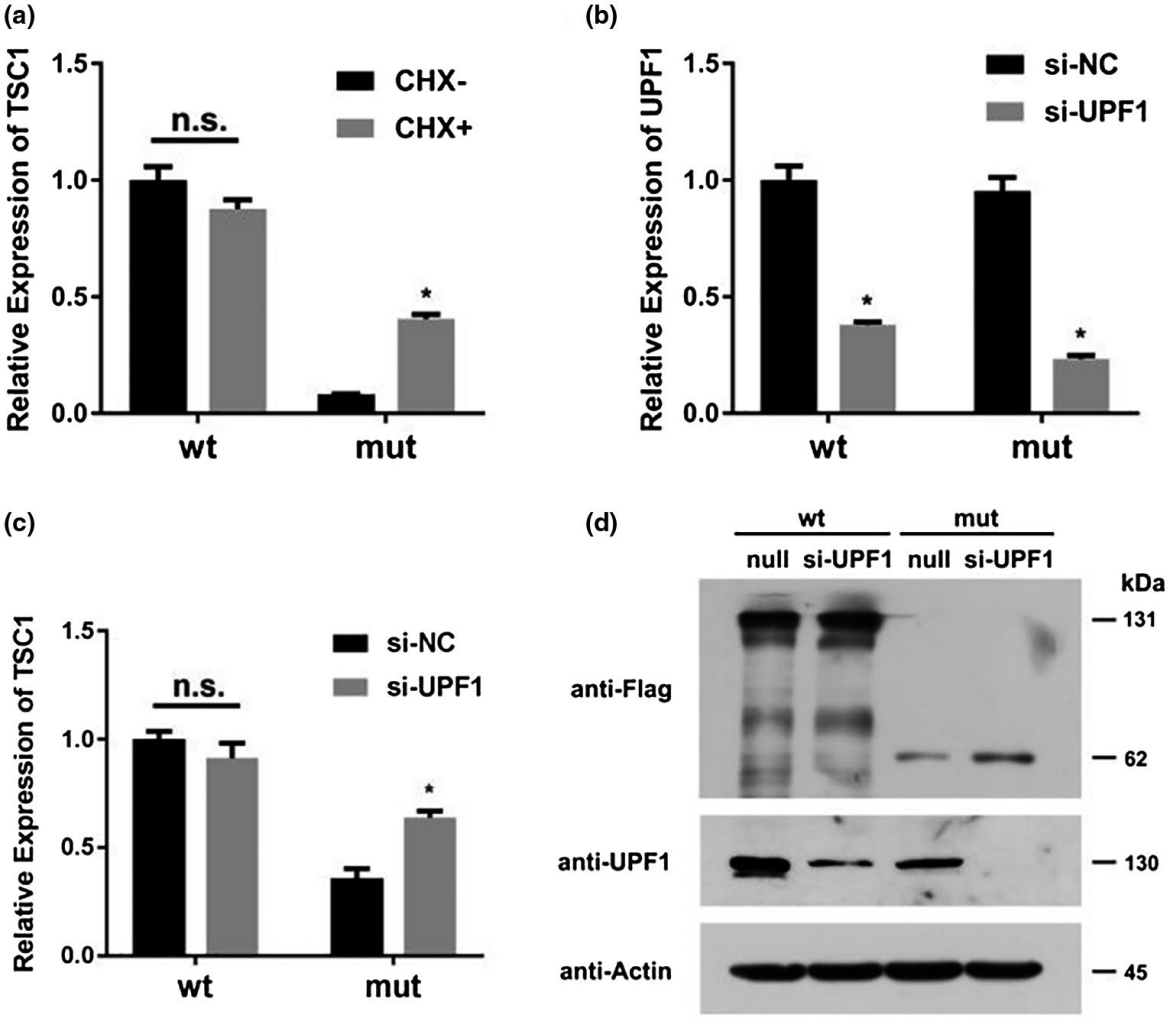
Effects of cycloheximide and UPF1 siRNA on pEGFP‐C1‐*TSC1* expression in transfected cells. (a) The level of pEGFP‐C1‐TSC1 mRNA transcribed from the cell transfected with mutant plasmid has significantly increased after treatment with Cycloheximide, but no difference in the pEGFP‐C1‐TSC1 mRNA level transcribed from the cell transfected with wt plasmid. (b) The expression levels of the UPF1 mRNA were all significantly decreased in transfected cells treated with UPF1 siRNA. (c) UPFI siRNA significantly increased mutant pEGFP‐C1‐TSC1 mRNA levels but made no difference to wt pEGFP‐C1‐TSC1. (d) Mutant pEGFP‐C1‐TSC1 proteins significantly increased after treatment with UPF1 siRNA but no difference in wt pEGFP‐C1‐TSC1 proteins

To find out whether NMD was associated with the lower levels of mutant pEGFP‐C1‐*TSC1* mRNAs, the wild‐type and mutant‐type plasmids were transiently transfected into 293T cells, and then, treated with cycloheximide CHX. The mRNA expression was analyzed by qPCR (Figure 10A). Cycloheximide might be subject to NMD which can inhibit translation and stabilize mutant mRNAs. It is an inhibitor of protein biosynthesis in eukaryotic organisms, exerting its effect by interfering with the translocation step in protein synthesis, thus, blocking translational elongation (Schneider‐Poetsch et al., 2010). The level of pEGFP‐C1‐*TSC1* mRNA transcribed from the cell transfected with mutant plasmid has significantly increased after treatment with Cycloheximide, but no difference in the pEGFP‐C1‐*TSC1* mRNA level transcribed from the cell transfected with wt plasmid. These results showed that cycloheximide inhibited NMD and protected the mutant pEGFP‐C1‐*TSC1* mRNAs from degradation.

NMD factor UPF1, which is the central regulator in NMD, function together with other UFP proteins to recruit decay enzymes to promote decay of mRNAs containing premature termination codons (PTCs) (Kervestin & Jacobson, 2012). UPF1 siRNA has been proved to be able to inhibit UFP1 expression and further inhibit the occurrence of NMD. After 48 hours of incubation, total RNA and proteins were extracted from HEK 293T cells transfected with UPF1 siRNA and a mutant or a wild‐type pEGFP‐C1‐*TSC1*, and then, analyzed by qPCR and Western Blot. The expression levels of the UPF1 mRNA were all significantly decreased in transfected cells treated with UPF1 siRNA (Figure 10B). In the cells transfected with mutant plasmid, the level of pEGFP‐C1‐*TSC1* mRNA has significantly increased after treatment with UPF1 siRNA compared with which without treatment with UPF1 siRNA. However, in the cells transfected with the wt plasmid, UPFI siRNA made no difference on pEGFP‐C1‐*TSC1* mRNA levels compared with cells without treatment of UPF1 siRNA (Figure 10C). The similar expression patterns of UPF1 and pEGFP‐C1‐*TSC1* proteins were observed via Western blot. These findings figured out that UPF1 siRNA specifically knocked down the amount of UPF1 protein, consequently inhibiting the occurrence of NMD (Figure 10D).

## 4 DISCUSSION

The genetic heterogeneity might correlate to the clinical variability among TSC‐affected families. About 75%–90% of the patients with definite diagnosis of TSC have identifiable mutations (Glushkova et al., 2018). The greatly lower frequency of novel mutations in *TSC1* versus *TSC2*, and *TSC1* disease is less severe than *TSC2* (Dabora et al., 2001). The most common type was frame‐shift mutations, whose proportion was 29.4% approximately (He et al., 2020). In this study, the patients symptom just has been involved in skin and brain features, but no tumorous lesions found in heart and liver, indicating the lower grade illness. We demonstrate two members from a Chinese family who suffered the same pathogenic mutation but showed different clinical manifestations, which consistent with the diversity in clinical features of the tuberous sclerosis complex. Gene test revealed a novel heterozygous frameshift mutation c.1550_1551del (p.Arg517GlnfsTer17) on Exon 15 of the *TSC1* in both patients. The mutation alter the intrinsic base sequence and introduce a premature stop codon, so we hypothesized the pathogenesis of this mutation might be associated with abnormal splicing and nonsense‐mediated mRNA degradation (NMD).

Alternative splicing is universal in eukaryotes and involves in nearly 95% of mammalian genes and various regulatory processes, playing a crucial role in hereditary disease and cancer. In pre‐mRNA, the splicing is governed by cis‐regulatory sequences. If a mutation causes an crucial exon splicing enhancers disruption, it may also be a pathogenic mutation, even though it does not change the encoded amino acid of a protein (Kornblihtt et al., 2013). The mutation detected from the family did not produce a new splicing pattern, but changed the proportion of the two original splicing modes. It indicated that the mutation did not produce a new splice site but changed the regulating factors. However, this requires further experimentation to verify.

Nonsense‐mediated mRNA decay (NMD) is an important RNA surveillance mechanism in eukaryotic cells. Once the UFP1 recognize mRNA containing premature termination codon (PTC), other NMD factors will be recruited to degrade the PTC‐containing mRNA. NMD activation also lead to dreamatical downregulation expression of the PTC‐containing allele, degradation of the nascent polypeptide chain, as well as inhibiting the pre‐mRNA splicing. In heterozygous carriers, normal allele express but PTC‐containing allele inactivated, causing the total level of functional proteins to be halved, which may cause disease due to haploinsufficiency (Kervestin & Jacobson, 2012; Lykke‐Andersen & Jensen, 2015). Our study revealed a decrease of protein level in mutant cells induced by NMD, suggesting that haploinsufficiency may be associated with the pathogenesis of this mutation.

Inactivation of either *TSC1* or *TSC2* results in decrease of TSC protein complexes and over activation of mTOR signaling transduction pathway, which further contributes to abnormal cell proliferation, differentiation, and other dysfunctions. The mTOR signaling pathway has been extensively studied and mTOR inhibitors have been approved as a therapeutic option for clinical use (Saffari et al., 2019). It is possible that complete loss of hamartin_the *TSC1* gene product, has different effects in cells, compared with the loss of tuberin, the *TSC2* gene product (Dabora et al., 2001). The two‐hits model for hamartoma development in TSC hypothesizes that second somatic mutations occur in the hamartomas cells. This model has been well proven by loss of heterozygosity (Green, Smith, & Yates, 1994; Henske et al., 1996).

We have identified the novel frameshift mutation c.1550_1551del (p.Arg517GlnfsTer17) on Exon 15 of *TSC1* gene. The hamartin were failure to be synthesized, led to hamartin‐tuberin heterodimer defect and dysfunction. So the mTOR signaling pathway was over activated, which caused their hamartoma and neuropsychiatric disorders. According with “Criteria for classifying pathogenic variants” of ACMG standards and guidelines (Richards et al., 2015), the |mutation c.1550_1551del meet one very strong evidence of pathogenicity, one strong evidences, two moderate evidences, and three supporting evidences for TSC. As far as we know, loss of function caused by frameshift mutation in *TSC1* is a kind of well‐known mechanism of TSC. Our study has verified the mutation has destructive effect on *TSC1* from models in vitro. Protein length changes as a result of stop‐loss variant. Multiple lines of computational evidence support deleterious effect on *TSC1*. So it is reasonable to suggest that the c.1550_1551del (p.Arg517GlnfsTer17) on Exon 15 of *TSC1* gene is a kind of pathogenic mutation.

However, the molecular mechanism by which mutations cause reduction of TSC‐associated proteins is still lacking. This study will further expand the *TSC* mutation database and, to some extent, increase the comprehension of the molecular mechanism of TSC pathogenesis. Our findings show immense potential for genetic counseling and prenatal diagnoses. We look forward to more discoveries of the pathogenesis at the transcription and translation levels in the future, thus, providing another therapeutic option for the disease.

## CONFLICT OF INTEREST

The authors declare that they have no conflicting interests.

## AUTHORS’ CONTRIBUTIONS

Cong Qiu did the data statistical analysis and drafted the manuscript. Chengyan Li collect the data and organized the figures. Xiaoyun Tong, Luoyang Dai, Wenda Liu, and Yulie Xie participated in the experiments. Qimei Zhang collected the patients’ samples. Guohua Yang generally supervised the research group and revised the manuscript. Tao Li participated in the design of the study and obtained the funding.

## Data Availability

The data that support the findings of this study are available from the corresponding author upon reasonable request.

## References

[mgg31410-bib-0001] Chomczynski, P. , & Sacchi, N. (2006). The single‐step method of RNA isolation by acid guanidinium thiocyanate‐phenol‐chloroform extraction: Twenty‐something years on. Nature Protocols, 1, 581–585. 10.1038/nprot.2006.83 17406285

[mgg31410-bib-0002] Crino, P. B. , Aronica, E. , Baltuch, G. , & Nathanson, K. L. (2010). Biallelic TSC gene inactivation in tuberous sclerosis complex. Neurology, 74, 1716–1723. 10.1212/WNL.0b013e3181e04325 20498439 PMC2882213

[mgg31410-bib-0003] Dabora, S. L. , Jozwiak, S. , Franz, D. N. , Roberts, P. S. , Nieto, A. , Chung, J. , … Kwiatkowski, D. J. (2001). Mutational analysis in a cohort of 224 tuberous sclerosis patients indicates increased severity of TSC2, compared with *TSC1*, disease in multiple organs. American Journal of Human Genetics, 68, 64–80. 10.1086/316951 11112665 PMC1234935

[mgg31410-bib-0004] Dibble, C. C. , Elis, W. , Menon, S. , Qin, W. , Klekota, J. , Asara, J. M. , … Manning, B. D. (2012). TBC1D7 Is a third subunit of the TSC1‐TSC2 complex upstream of mTORC1. Molecular Cell, 47, 535–546. 10.1016/j.molcel.2012.06.009 22795129 PMC3693578

[mgg31410-bib-0005] Glushkova, M. , Bojinova, V. , Koleva, M. , Dimova, P. , Bojidarova, M. , Litvinenko, I. , … Todorova, A. (2018). Molecular genetic diagnostics of tuberous sclerosis complex in Bulgaria: Six novel mutations in the *TSC1* and *TSC2* genes. Journal of Genetics, 97, 419–427. 10.1007/s12041-018-0927-7 29932062

[mgg31410-bib-0006] Green, A. J. , Smith, M. , & Yates, J. R. W. (1994). Loss of heterozygosity on chromosome 16p 13.3 in hamartomas from tuberous sclerosis patients. Nature Genetics, 6, 193–196. 10.1038/ng0294-193 8162074

[mgg31410-bib-0007] He, J. , Zhou, W. , Shi, J. , Lin, J. , Zhang, B. , & Sun, Z. (2020). *TSC1* and *TSC2* gene mutations in chinese tuberous sclerosis complex patients clinically characterized by epilepsy. Genetic Testing and Molecular Biomarkers, 24, 1–5. 10.1089/gtmb.2019.0094 31855466

[mgg31410-bib-0008] Henske, E. P. , Scheithauer, B. W. , Short, M. P. , Wollmann, R. , Nahmias, J. , Hornigold, N. , … Kwiatkowski, D. J. (1996). Allelic Loss is frequent in tuberous sclerosis kidney lesions but rare in brain lesions. American Journal of Human Genetics, 59(2), 400–406. 8755927 PMC1914733

[mgg31410-bib-0009] Johnson, M. W. , Kerfoot, C. , Bushnell, T. , Li, M. , & Vinters, H. V. (2001). Hamartin and tuberin expression in human tissues. Modern Pathology, 14, 202–210. 10.1038/modpathol.3880286 11266527

[mgg31410-bib-0010] Kervestin, S. , & Jacobson, A. (2012). NMD: A multifaceted response to premature translational termination. Nature Reviews Molecular Cell Biology, 13, 700–712. 10.1038/nrm3454 23072888 PMC3970730

[mgg31410-bib-0011] Kornblihtt, A. R. , Schor, I. E. , Alló, M. , Dujardin, G. , Petrillo, E. , & Muñoz, M. J. (2013). Alternative splicing: A pivotal step between eukaryotic transcription and translation. Nature Reviews Molecular Cell Biology, 14, 153–165. 10.1038/nrm3525 23385723

[mgg31410-bib-0012] Lykke‐Andersen, S. , & Jensen, T. H. (2015). Nonsense‐mediated mRNA decay: An intricate machinery that shapes transcriptomes. Nature Reviews Molecular Cell Biology, 16, 665–677. 10.1038/nrm4063 26397022

[mgg31410-bib-0013] Mi, C. R. , Wang, H. , Jiang, H. , Sun, R. P. , & Wang, G. X. (2014). Mutation screening of *TSC1* and *TSC2* genes in Chinese Han children with tuberous sclerosis complex. Genetics and Molecular Research, 13, 2102–2106. 10.4238/2014.March.24.14 24737435

[mgg31410-bib-0014] Northrup, H. , Krueger, D. A. , Northrup, H. , Krueger, D. A. , Roberds, S. , Smith, K. , … Frost, M. D. (2013). Tuberous sclerosis complex diagnostic criteria update: Recommendations of the 2012 international tuberous sclerosis complex consensus conference. Pediatric Neurology, 49, 243–254. 10.1016/j.pediatrneurol.2013.08.001 24053982 PMC4080684

[mgg31410-bib-0015] Richards, S. , Aziz, N. , Bale, S. , Bick, D. , Das, S. , Gastier‐Foster, J. , … Rehm, H. L. (2015). Standards and guidelines for the interpretation of sequence variants: A joint consensus recommendation of the American College of Medical Genetics and Genomics and the Association for Molecular Pathology. Genetics in Medicine, 17(5), 405–424. 10.1038/gim.2015.30 25741868 PMC4544753

[mgg31410-bib-0016] Rosner, M. , Hofer, K. , Kubista, M. , & Hengstschläger, M. (2003). Cell size regulation by the human TSC tumor suppressor proteins depends on PI3K and FKBP38. Oncogene, 22, 4786–4798. 10.1038/sj.onc.1206776 12894220

[mgg31410-bib-0017] Saffari, A. , Brösse, I. , Wiemer‐Kruel, A. , Wilken, B. , Kreuzaler, P. , Hahn, A. , … Hethey, S. (2019). Safety and efficacy of mTOR inhibitor treatment in patients with tuberous sclerosis complex under 2 years of age – A multicenter retrospective study. Orphanet Journal of Rare Diseases, 14, 1–13. 10.1186/s13023-019-1077-6 31053163 PMC6500021

[mgg31410-bib-0018] Schneider‐Poetsch, T. , Ju, J. , Eyler, D. E. , Dang, Y. , Bhat, S. , Merrick, W. C. , … Liu, J. O. (2010). Inhibition of eukaryotic translation elongation by cycloheximide and lactimidomycin. Nature Chemical Biology, 6, 209–217. 10.1038/nchembio.304 20118940 PMC2831214

[mgg31410-bib-0019] Tee, A. R. , Fingar, D. C. , Manning, B. D. , Kwiatkowski, D. J. , Cantley, L. C. , & Blenis, J. (2002). Tuberous sclerosis complex‐1 and ‐2 gene products function together to inhibit mammalian target of rapamycin (mTOR)‐mediated downstream signaling. Proceedings of the National Academy of Sciences of the United States of America, 99, 13571–13576. 10.1073/pnas.202476899 12271141 PMC129715

[mgg31410-bib-0020] The European Chromosome 16 Tuberous Sclerosis Consortium . (1993). Identification and characterization of the tuberous sclerosis gene on chromosome 16. Cell, 75, 1305–1315. 10.1016/0092-8674(93)90618-Z 8269512

[mgg31410-bib-0021] Van Slegtenhorst, M. , De Hoogt, R. , Hermans, C. , Nellist, M. , Janssen, B. , Verhoef, S. et al (1997). Identification of the tuberous sclerosis gene *TSC1* on chromosome 9q34. Science (80‐.), 277, 805–808. 10.1126/science.277.5327.805 9242607

[mgg31410-bib-0022] Van Slegtenhorst, M. , Nellist, M. , Nagelkerken, B. , Cheadle, J. , Snell, R. , Van Den Ouweland, A. , … van der Sluijs, P. (1998). Interaction between hamartin and tuberin, the *TSC1* and *TSC2* gene products. Human Molecular Genetics, 7, 1053–1057. 10.1093/hmg/7.6.1053 9580671

